# Immunomodulation targeting of both Aβ and tau pathological conformers ameliorates Alzheimer’s disease pathology in TgSwDI and 3xTg mouse models

**DOI:** 10.1186/1742-2094-10-150

**Published:** 2013-12-13

**Authors:** Fernando Goñi, Krystal Herline, Daniel Peyser, Kinlung Wong, Yong Ji, Yanjie Sun, Pankaj Mehta, Thomas Wisniewski

**Affiliations:** 1 Department of Neurology, New York University School of Medicine, 550 First Avenue, New York, NY 10016, USA; 2 Department of Pathology, New York University School of Medicine, 550 First Avenue, New York, NY 10016, USA; 3 Department of Psychiatry, New York University School of Medicine, 550 First Avenue, New York, NY 10016, USA; 4 New York State Institute for Basic Research in Developmental Disabilities, 1050 Forest Hill Rd., Staten Island, NY 10314, USA; 5 Department of Neurology, Tianjin Huanhu Hospital, Tianjin, China; 6 New York University School of Medicine, Alexandria ERSP, Rm 802, 450 East 29th Street, New York, NY 10016, USA

**Keywords:** Alzheimer’s disease, British amyloidosis, β-sheet conformation, amyloid, vaccination, transgenic mice

## Abstract

**Background:**

Central to the pathogenesis of Alzheimer’s disease (AD) and many other neurodegenerative diseases is the conformational change of a normal self-protein into toxic oligomeric species and amyloid deposits. None of these disorders have an effective therapy, but immunization approaches hold great promise. We have previously shown that active immunization with a novel peptide when polymerized into a stable oligomeric conformation, pBri, induced a humoral immune response to toxic Aβ species in an AD model, APP/PS1 transgenic (Tg) mice, reducing plaque deposits. pBri is a glutaraldehyde polymerized form of the carboxyl fragment of an amyloidogenic protein, which is deposited in the brains of patients with a rare autosomal dominant disease due to a missense mutation in a stop codon, resulting in the translation of an intronic sequence, with no known sequence homology to any mammalian protein.

**Methods:**

In the current study we tested whether pBri-peptide-based immunomodulation is effective at reducing both vascular amyloid deposits and tau-related pathology using TgSwDI mice with extensive congophilic angiopathy and 3xTg mice with tau pathology.

**Results:**

Our results indicate that this immunomodulation approach, which produces a humoral response to proteins in a pathological conformation, is effective at reducing both Aβ and tau-related pathologies.

**Conclusions:**

This immunomodulatory approach has the advantage of using a non-self-immunogen that is less likely to be associated with autoimmune toxicity. Furthermore we found that it is able to target all the cardinal features of AD concurrently.

## Introduction

Alzheimer’s disease (AD) is the most common cause of dementia globally, affecting approximately 36 million people currently and approximately 115 million by 2050 [1]. Presently available treatments have minimal or no effect on the course of the disease. Numerous novel therapeutic strategies are being developed, with active and passive immunization being among the most advanced approaches [2-6]. The neuropathology of AD consists of the accumulation of amyloid β (Aβ) as amyloid plaques and congophilic amyloid angiopathy (CAA), as well as the accumulation of aggregated, phosphorylated tau as neurofibrillary tangles [7]. The most toxic species of Aβ and aggregated tau are thought to be oligomeric, with both of these pathologies potentially spreading via extracellular soluble oligomers in a prion-like mechanism [8-10]. Aβ and tau oligomers, as well as amyloid plaques and neurofribrillary tangles (NFTs), share many structural and biophysical properties, such as a high β-sheet content, resistance to proteolytic degradation and neuronal toxicity. It has also been shown that Aβ and tau-related pathology can, under some conditions, seed or propagate each other.

Immunotherapy is very effective for both AD and prion diseases, at least in mouse models [11,12]. Unfortunately, two recent phase III trials of passive immunization for AD failed to achieve benefit by their primary end points [13]. To develop a highly successful immunotherapy for AD patients, several problems need to be overcome including: avoiding excessive cell-mediated immunity, which has been linked to autoimmune encephalitis; reducing CAA deposition without inducing associated microhemorrhages and/or vasogenic edema; reducing tau-related pathology, which is known to correlate with clinical status better than amyloid deposits, and targeting of oligomeric species, which are the most toxic [3]. To concurrently address all these issues we sought to develop therapeutic immunomodulation through a novel active immunization approach that specifically targets the pathological β-sheet conformation that is shared by Aβ and tau-disease-associated species. We used a polymerized British amyloidosis (pBri) related peptide in a predominantly β-sheet, in an oligomeric form, prepared using glutaraldehyde in a controlled manner. British amyloidosis (ABri) is a rare form of familial human amyloidosis associated with a missense mutation in a stop codon. This mutation results in the transcription of an intronic sequence, leading to the production of a highly amyloidogenic protein with a carboxyl terminus that has no sequence homology to any other native human protein, including Aβ and tau [14-16].

We hypothesized that through conformational mimicry the polymerized oligomeric pBri peptide, which corresponds to the carboxyl terminus of ABri, could induce a conformation-selective immune response that will recognize Aβ, as well as aggregated phosphorylated tau. Such an immunostimulatory approach would have a reduced risk of inducing autoimmune complications as it is specific to pathological conformers and the immunogen has no sequence homology to any known mammalian protein or peptide. In a prior study we tested this approach in an APP/PS1 AD mouse model with extensive amyloid plaque deposition [16] and evaluated for behavioral benefits and reductions in Aβ-related pathology histologically and biochemically. We showed that this immunomodulation targeting a pathological conformation of Aβ was highly effective at reducing amyloid plaques, correlating with behavioral rescue. The polyclonal antibody response obtained in the vaccinated mice specifically recognized plaques and dystrophic neurites in human brain tissue [16]. This novel therapeutic approach was commented on as a promising new direction for AD vaccination [17]. In the current study we addressed the critical issues of whether this type of approach could be used for both CAA and tau-related pathology, without any associated toxicity. To assess for effects on vascular amyloid and NFT-related pathology we used TgSwDI mice, which are a well-characterized model of CAA, and 3xTg mice with tau pathology, respectively [18,19]. We hypothesized that for immunomodulation to be highly successful it needs to target all the cardinal lesions of AD concurrently, that is amyloid plaques, CAA and NFT. Furthermore, to reduce the possibility of autoimmune complications, the immunogen needs to be a non-self-antigen in a conformation that induces an immune response that specifically targets pathological conformers. The evaluation of the effectiveness of our novel immunomodulatory approach in a variety of AD mouse models, which mimic different aspects of the human disease, is a critical step before any clinical testing.

## Materials and methods

### Synthesis of peptide, polymerization of the Bri peptide and assessment of conformation

This was done as previously published [16]. In brief, the 13 residue peptide corresponding to the carboxyl terminus of ABri (Cys-Ser-Arg-Thr-Val-Lys-Lys-Asn-Ile-Ile-Glu-Glu-Asn) was synthesized on an ABI 430A peptide synthesizer (AME Bioscience, Chicago, IL) at the Keck peptide synthesis facility at Yale University, CT. Mass spectroscopy of the lyophilized end-product was used to verify the expected molecular weight. To make the 13-residue Bri peptide immunogenic and to have a stable oligomeric conformation, the peptide was subjected to controlled polymerization using the following protocol.

The peptide was dissolved at 3 mg/ml, in 100 mM borate buffer saline (BBS), pH 7.4. Fresh 1% glutaraldehyde in BBS was prepared and added to the peptide to a final 5 mM glutaraldehyde concentration and incubated in an Eppendorf block shaker at 800 rpm and 56°C for 16 h. The solution was then quenched with 0.5 M glycine to make the solution 100 mM in glycine. After 5 min, the solution was diluted 1:3 with BBS, dialyzed against 2 mM BBS overnight at 4°C, aliquoted and lyophilized. To determine the degree of aggregation the original monomeric Bri peptide and polymerized Bri (pBri) were electrophoresed on 12.5% sodium dodecyl sulfate (SDS)-polyacrylamide tris-tricine gels under reducing conditions. Western blots were performed with a rabbit anti-ABri polyclonal Ab [16] (1:1,000 dilution). The secondary antibody (1:2,000 dilution) was peroxidase-linked anti-rabbit IgG (Amersham Biosciences, Piscataway, NJ), and the immunoreactive material was visualized as chemoluminescence on X-ray film with the enhanced chemiluminescence (ECL) detection kit (Pierce). For electron microscope studies, the original and polymerized Bri peptides were incubated at 1 mg/ml in phosphate-buffered saline, pH 7.4. Next, 3 μl of sample was put onto a carbon-coated 400 mesh Cu/Rh grid (Ted Pella, Inc, Redding, CA) and stained with 1% uranyl acetate in distilled water (Polysciences, Inc, Warrington, PA), as previously published [16]. For secondary structure analysis, aliquots of the original Bri peptide and pBri were reconstituted in 5 mM tris buffer (pH 7.0) to obtain a peptide concentration of 100 μM. Circular dichroism was measured on a Jasco J-720 spectropolarimeter (Easton, MD) equipped with a model CTC-344 circular temperature control system (Neslab Inc, Newington, NH).

### Immunization of mice

Animal studies were approved by the New York University School of Medicine Institutional Animal Care and Use Committee and were consistent with the recommendations of the American Veterinary Association. Mice facilities were under a strict 12 h light/dark cycle. Triple transgenic (3xTg) mice (PS1M146V, tauP301L and APPK670N/M671L) are a model of combined tau- and amyloid-related pathology [19,20]. To model vascular amyloid deposition, the well-characterized TgSwDI mouse model was used [21,22]. This model expresses the APPE693Q/D694N (Dutch and Iowa) and APPK670N/M671L (Swedish) mutations. The pBri peptide was dissolved in sterile saline at 1 mg/ml and mixed 4:1 with aluminum hydroxide (alum) adjuvant (Brenntag Biosector, Denmark). Each mouse received a weekly subcutaneous injection of 100 μl of the preparation (50 μg of pBri) for 4 weeks, starting at the age of 3 months, followed by an inoculation a month later and two subsequent bimonthly injections. The last three inoculations used 25 μg of pBri per animal and the ratio of saline to alum was changed to 9:1. Both the 3xTg and TgSwDI mice were divided into two groups of 14 mice each. There were equal numbers of males and females in each experimental group. For each transgenic line, one group received pBri immunization while the second control group received vehicle injections (alum alone). The mice were subject to behavioral testing at the age of 15 to 16 months.

### Sensorimotor and cognitive testing

Sensorimotor and cognitive testing were done as previously described [16,20,22]. Prior to testing, the mice were adapted to the room with lights on for 15 min. The main objective of performing these sensorimotor tasks was to verify that any treatment-related effects observed in the cognitive tasks could not be explained by differences in sensorimotor abilities.

#### Locomotor activity

A Hamilton-Kinder Smart-frame Photobeam System was used to make a computerized recording of animal activity over a designated period of time. Exploratory locomotor activity was recorded in a circular open-field activity chamber measuring 70 cm in diameter. A video camera mounted above the chamber automatically recorded horizontal movements in the open field in each dimension. Total distance was measured in centimeters traveled and is defined as the sum of sequential movement between interruptions of the animal. The duration of the behavior was timed over 15 min. Results were reported based on distance traveled (cm), mean resting time and maximum speed of the animal.

#### Traverse beam

This task tests balance and general motor coordination and function integration. Mice were assessed by measuring their ability to traverse a graded narrow wooden beam to reach a goal box. This specifically examines hind limb function. The mice were placed on a 1.1 cm wide beam, 50.8 cm long, supported 30 cm above a padded surface on two identical columns. A shaded goal box was attached at each end of the beam. Mice were placed on the beam in a perpendicular orientation to habituate, and were then monitored for a maximum of 60 sec. The number of foot slips each mouse made before falling or reaching the goal box was recorded for each of three successive trials. The average number of foot slips for all three trials was calculated and recorded. Errors are defined as foot slips and were recorded both numerically and using Feeney scores. To prevent injury from falling, a soft foam cushion was always kept underneath the beam. Animals that fell off were placed back in the position they were in prior to the fall.

#### Rotarod

Each animal was placed onto a rod (diameter 3.6 cm) apparatus to assess differences in motor coordination and balance by measuring fore- and hindlimb motor coordination and balance (Rotarod 7650 accelerating model; Ugo Basile, Biological Research Apparatus, Varese, Italy). This was designed to assess motor behavior without a practice confound. The animals were habituated to the apparatus by receiving training sessions of two trials, which was sufficient to reach a baseline level of performance. The mice were tested a further three times, with increasing speed. During habituation, the rotor rod was set at 1.0 rpm, which was gradually increased every 30 sec to a maximum speed of 40.0 rpm. The rod was wiped clean with 30% ethanol solution after each session. A soft foam cushion was placed beneath the apparatus to prevent potential injury from falling. Each animal was tested for three sessions, with each session separated by 15 min. The latency to fall or invert (by clinging) from the top of the rotating barrel was recorded.

#### Radial arm maze

Spatial learning was evaluated using an eight-arm radial maze with a water well at the end of each arm, as we have previously published [16,20,22], for both TgSwDI and 3xTg mice. For each group of transgenic mice, an age-matched control group of non-transgenic mice (*n* = 10) were also tested in the radial arm maze. Clear Plexiglas guillotine doors, operated by a remote pulley system, controlled access to the arms from a central area from which the animals entered and exited the apparatus. After 4 days of adaptation to the maze, water-restricted mice (only given access to water 2 h per day) were given one training session per day for 10 consecutive days. We use this relatively long adaptation period as we have found that these transgenic (Tg) AD mice tend to be anxious and will not run the maze well without adaptation [16,20,22]. Prior to each day’s testing, the mice were adapted to the room with the lights on for 15 min. For each session, all arms were baited with saccharine-flavored water, and the animals were permitted to enter all arms until the eight rewards had been consumed. The number of errors (entries to previously visited arms) and time to complete each session were recorded.

#### Antibody levels

Antibody levels were determined in duplicate on 1:100 dilutions of plasma using ELISA as described previously [16,20,22], in which 50 μg/plate aggregated Aβ1-42, aggregated Aβ1-40, pBri or purified human paired helical filaments (PHF) was coated onto Immulon 2HB 96-well microtiter wells (Thermo, Waltham, MA). The human PHF was prepared and characterized for purity by Western blotting with PHF1 and electron microscopy, as previously published [16]. The bound antibodies were detected by a horseradish peroxidase-labeled goat anti-mouse IgG (Amersham Biosciences, Piscataway, NJ) or a peroxidase conjugated goat anti-mouse IgM (Sigma; A8786). The color developing substrate was tetramethylbenzidine (TMB) (Pierce, Rockford, IL) and the readings were made at 450 nm.

### Histology

The mice were anesthetized with sodium pentobarbital (150 mg/kg, intraperitoneally), perfused transaortically with phosphate buffer, and the brains processed as described previously [16,20,22]. The right hemisphere was immersion-fixed in periodate-lysine-paraformaldehyde, whereas the left hemisphere was snap-frozen for measurements of Aβ levels in both transgenic lines and phosphorylated tau in the 3xTg mice. Serial coronal sections (40 μm) were cut (30 to 40 sections in total), and every fifth section was stained with a mixture of 4G8 and 6E10, which are monoclonal antibodies that recognize Aβ and stain both pre-amyloid and Aβ plaques [23,24]. In 3xTg mice, the degree of tau-related pathology was determined with anti-abnormally phosphorylated tau monoclonal antibodies PHF1 (which recognizes phosphorylated serine in position 396 and 404) [25] and AT8 (which recognizes tau phosphorylated at both serine 202 and threonine 205) [26]; these antibodies were kindly provided by Dr Peter Davies from the Albert Einstein College of Medicine, Bronx, NY.

Glial fibrillary acidic protein (GFAP) is a component of the glial intermediate filaments that forms part of the cytoskeleton and is found predominantly in astrocytes. CD45 is a protein tyrosine phosphatase, commonly used to detect microglial activation at the later stages of plaque development [27,28]. Two series of sections were immunostained with anti-GFAP (Dako, Carpinteria, CA) and anti-CD45 (Abd Serotec, Raleigh, NC) antibodies.

Immunostaining was performed as described previously [16,20,22]. Briefly, sections were incubated in 6E10/4G8 each at a 1:1000 dilution in PBS-T for 3 h. A mouse-on-mouse immunodetection kit (Vector Laboratories, Burlingame, CA) was used. The sections were incubated first with biotinylated anti-mouse IgG secondary antibody for 1 h at a 1:2000 dilution and later with the avidin-peroxidase complex for 30 min at the same dilution. The sections were then reacted in 3,3-diaminobenzidine tetrahydrochloride with nickel ammonium sulfate (Mallinckrodt, Paris, KY) color intensification solution. Immunohistochemistry of 6E10/4G8 immunolabeled tissue sections was quantified with a Bioquant image analysis system (BIOQUANT Image Analysis Corporation, Nashville, TN), and unbiased sampling was used [24]. All procedures were performed by an individual blinded to the experimental conditions of the study.

The cortical area analyzed was dorsomedial from the cingulate cortex and extended ventrolaterally to the rhinal fissure within the right hemisphere. The area of the grid was 800 × 800 μm^2^, and the Aβ deposit load was measured for 20 cortical frames per mouse (640 × 480 μm^2^ each) chosen randomly. The threshold of the Aβ immunoreactive areas was set so that areas of <5 μm in diameter are not counted. This was done so that small artifactual areas of staining were not counted and the intra-neuronal immunoreactivity was also not counted. With the latter caveat, the Aβ burden was defined as the percentage of the area in the measurement field occupied by the reaction product. GFAP-staining (polyclonal, 1:1000; 3 h, Dako, Denmark) was performed with a primary antibody diluent composed of 0.3% triton X-100, 0.1% sodium azide, 0.01% bacitracin, 1% bovine serum albumin (BSA) and 10% normal goat serum in PBS, and a secondary biotinylated goat anti-rabbit antibody (Vector Laboratories, Burlingame, CA) reacted for 1 h at 1:1000 dilution. The analysis of the CD45 immunohistochemistry (rat anti-mouse, 1:500; 3 h, Serotec) was performed like the GFAP-staining except that the secondary antibody was goat anti-rat (Vector Laboratories, Burlingame, CA) diluted 1:1000.

The tau burden was analyzed based on the extent of immunostaining with two anti-tau antibodies, PHF1 and AT8, as previously described [20]. PHF1 was analyzed at 10× magnification in both the hippocampus and cortex. Approximately six sections were analyzed per animal. AT8 was analyzed at 10× magnification for the hippocampus (AT8 was not analyzed in the cortex as the cortical AT8 immunolabeling was minimal). Approximately eight cortical sections and six hippocampal sections were analyzed per animal. The rating was based on the number of reactive neuronal bodies and processes. Reactive astrocytosis (GFAP immunoreactivity) was rated on a scale of 0 to 4. The rating was based on a semiquantitative analysis of the extent of GFAP immunoreactivity (the number of GFAP immunoreactive cells and complexity of astrocytic branching), as we have previously published [16,20,22]. The assessment of the CD45 immunostained sections was based on a semiquantitative analysis of the extent of microgliosis (0, no microglia; 1, a few resting microglia; 2, a few ramified and/or phagocytic microglia; 3, moderate number of ramified/phagocytic microglia; 4, numerous ramified/phagocytic microglia), as we have previously reported [16,20,22].

Perl’s iron staining was performed for the TgSwDI mice on another set of sections to detect cerebral microhemorrhages, as we have previously reported [22]. Sections were stained in a solution containing 5% potassium ferrocyanide and 10% hydrochloric acid for 40 min.

### Tissue homogenization and sandwich ELISA assay for soluble Aβ levels

Aβ was extracted from brain tissue as described [24] for both TgSwDI and 3xTg mice. Brains were weighed and homogenized (20% w/v) in a homogenization buffer, 20 mM Tris, 250 mM sucrose, 1 mM Ethylenediaminotetraacetic acid disodium salt (EDTA), 1 mM Ethylene glycol tetraacetic acid tetrasodium salt (EGTA) with freshly prepared 100 mM phenylmethylsulfonyl fluoride, 5 μg/ml pepstatin A and a protease inhibitor cocktail (Complete, Roche Diagnostics, Indianapolis, IN). Subsequently, 400 μl of the solutions was spun at 100,000 *g* for 1 h at 4°C, aliquoted, flash-frozen on dry ice and stored at -80°C until used for both Aβ and tau measurements in ELISA and Western blots.

The total and soluble Aβ levels were measured using a combination of mouse monoclonal antibody 6E10 (specific to an epitope present on amino acid residues 1 to 16 of Aβ) and two different rabbit polyclonal antibodies specific for Aβ40 (R162) and Aβ42 (R165), in a double-antibody sandwich ELISA as described previously [23,24]. The optical density (OD) was measured at 450 nm. The relation between OD and Aβ peptide concentration was determined by a four-parameter logistic log function. Non-linear curve fitting was performed with the KinetiCalc program (Biotek Instruments, Inc, Winooski, VT) to convert the OD of plasma to estimated concentrations. The assay was performed by an investigator blinded to group assignment. The levels of Aβ species are presented as micrograms of Aβ per gram of wet brain, taking into account dilution factors introduced by multiple steps throughout the assay (brain homogenization and extraction procedures).

### Western blot and Meso Scale Discovery electrochemiluminescence analysis of phosphorylated tau for 3xTg mice

For Western immunoblot analysis, 20% w/v brain homogenates from the 3xTg mice were centrifuged at 25,000 *g* for 10 min at 4°C, and the supernatants were transferred to clean tubes and stored as previously described [23,24]. The total protein concentration in the supernatant was determined using the bicinchoninic acid assay (Pierce, Rockford, IL). Samples (40 μg of total protein) were mixed with an equal volume of tricine sample buffer (BioRad, Hercules, CA), electrophoresed on 12.5% SDS-tris-tricine polyacrylamide gels under non-reducing conditions and transferred to nitrocellulose membranes. To assess whether there was equal protein loading in each lane, the membranes were stained with reversible 0.1% Fast Green FCF (Fisher Scientific, USA) in 25% methanol destained in 25% methanol and transferred to distilled water. The blots were then blocked with 5% non-fat dry milk in 50 mM tris buffer saline- 0.1% Tween 20 (TBS-T), pH 8.3, for 2 h at room temperature, then incubated with PHF1 diluted 1:500 in TBS-T, 0.1% BSA for 2 h at room temperature. Bound antibodies were detected after 1 h incubation with horseradish peroxidase-conjugated goat anti-mouse IgG 1:8000 (Pierce, Rockford, IL) and the ECL detection system (Pierce, Rockford, IL). The specificity of the PHF1 band was confirmed by Western blots using homogenates of non-transgenic mouse brains. Only the bands detected by PHF1 in 3xTg transgenic mouse homogenates and not in the non-transgenic mouse homogenates were quantitated. Densitometric analysis of the PHF1 specific bands was performed with the NIH Image J software version 1.34.

The same 20% brain homogenates containing only soluble forms of tau were centrifuged at 100,000 *g*. Total tau and phosphorylated tau (Thr231) were quantified using a Meso Scale Discovery (MSD) (Rockville, MD) system that utilizes electrochemiluminescence analysis. The multi-array phospho (Thr231) tau assay kit, which measures both total human tau and phosphorylated Thr231 tau, was used with the MesoQuickPlex SQ 120 system, following the manufacturer’s instructions. In brief, samples were diluted 1:125 with the provided standard diluent buffer and 100 μl aliquots were seeded in each well. The plates were incubated for 2 h at room temperature, then washed four times for 25 sec each, before the Hu Aggregated Aβ biotin conjugate was added and incubated for 1 h. The plates were again washed four times and the Streptavidin-HRP working solutions was added. The plates were covered to block the light and incubated for 25 min before the reaction was stopped with the stop solution. The plates were read on the MSD system at 450 nm. All data were recorded and calculations made using the software provided with the MSD system.

### Quantitation of aggregated/oligomeric Aβ

Aggregated/oligomeric Aβ levels were determined using the Human Aggregated Aβ ELISA kit (Invitrogen, Camarillo, CA), following the manufacturer’s instructions. In brief, the levels of aggregated/oligomeric Aβ in each sample were measured against a standard containing aggregated Aβ. Next 20% w/v brain homogenates were thawed, diluted 1:4 with the diluents buffer and applied to the ELISA plates. The samples were then incubated for 2 h at room temperature allowing the N-terminal portion of the Aβ to bind the pre-coated capture monoclonal antibody, followed by extensive washing and incubation for 1 h at room temperature with biotin conjugated detection antibodies (same as the capture antibody), which binds only to the immobilized aggregated Aβ. After removal of excess antibody, horseradish peroxidase-labelled streptavidin was added. The sample was incubated for 30 min, followed by washing and TMB substrate incubation to produce color. The intensity of this colored product is directly proportional to the concentration of aggregated/oligomeric Aβ in the sample. The standards produced a linear curve and the best-fit lines determined by linear regression were used to calculate aggregated Aβ concentrations in the samples.

### Statistical analysis

Data from the accelerating rotor rod and locomotor test were analyzed using two-tail, Student’s *t*-tests. The data collected from the radial arm maze test was analyzed using two-way ANOVA, and also by one-way ANOVA followed by Newman–Keuls *post hoc* tests. Differences in total amyloid burden, levels of extracted Aβ, levels of Aβ aggregates/oligomers, tau burden, astrogliosis and microgliosis between the two groups were analyzed using two-tailed *t*-tests. All statistical tests were performed using Prism 6.0 (Graphpad, San Diego, CA).

## Results

### Antibody titers for Aβ40, Aβ42, pBri and human purified PHF

**Figure 1:**
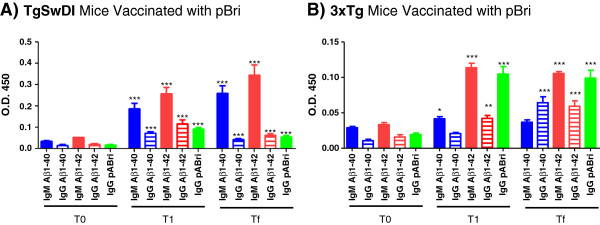
**Plasma antibody levels to aggregated Aβ40, Aβ42 and pBri in pBri-vaccinated mice.** Shown are bar graphs of the IgM and IgG antibody levels of **(A)** TgSwDI and **(B)** 3xTg and against Aβ40, Aβ42 and pBri at T0 (before vaccination), T1 (after the fourth inoculation) and Tf (at the time of sacrifice). Plasma samples were evaluated at a dilution of 1:100 in duplicate. (*P ≤ 0.05, **P ≤ 0.01 and ***P ≤ 0.001 by unpaired two-tailed *t*-tests, comparing values at T1 and Tf to T0. Error bars represent mean ± standard error of the mean). Aβ, amyloid β; OD, optical density.

For both the TgSwDI strain (Figure 1A) and the 3xTg strain (Figure 1B), pBri-vaccinated mice showed significant IgG and IgM titers at T1 (after the fourth inoculation) and Tf (at the time of sacrifice) against aggregated Aβ40, aggregated Aβ42 and polymerized Bri, compared to equivalent T0 titers (prior to vaccination) by two-tailed unpaired *t*-tests. In the control groups for both TgSwDI and 3xTg strains, as expected, there were no significant differences in T1 and Tf titers against Aβ40, Aβ42 and pBri versus T0, with OD values all being ≤0.03 (data not shown).

**Figure 2:**
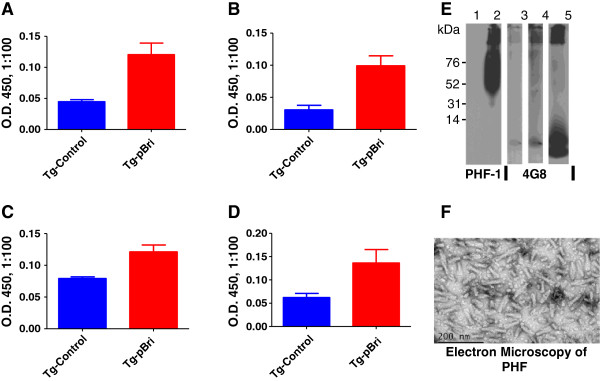
**Antibody titers in plasma reactive against purified human paired helical filaments (PHF) at Tf and characterization of the PHF preparation.** Titers for the TgSwDI and 3xTg mice were determined against human purified PHF at Tf for both IgM and IgG and were compared to vehicle-inoculated Tg controls. Shown are the titers for TgSwDI IgM **(A)**, TgSwDI IgG **(B)**, 3xTg IgM **(C)** and 3xTg IgG **(D)**. These differences in titer are statistically significant by two-way unpaired *t*-tests (*P* = 0.0038, *P* = 0.0037, *P* = 0.0057 and *P* = 0.04, respectively). **(E)** Western blot of the human PHF sample used to seed the ELISA plates in (**A) to (D). (F)** Electron micrograph of the human PHF sample used, revealing the expected paired helical filaments. OD, optical density; Tg, transgenic.

**Figure 3:**
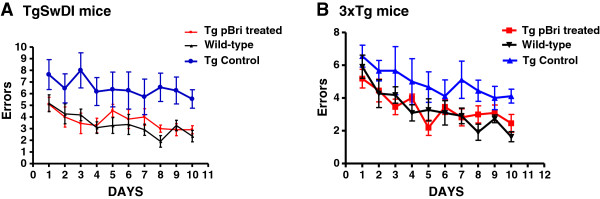
**Cognitive testing of TgSwDI and 3xTg mice using a radial arm maze.** The number of errors is plotted versus the day of testing. **(A)** Results for TgSwDI mice. **(B)** Results for 3xTg mice. There are no differences between the wild-type controls and the pBri-treated TgSwDI mice. There are statistically significant differences between the vehicle control mice and the pBri-treated TgSwDI mice and wild-type controls using two-way ANOVA, and the treatment effect had *P* < 0.001. Newman–Keuls *post hoc* analyses indicate that both the wild-type controls and pBri-vaccinated Tg mice were significantly different from the control Tg mice (*P* < 0.001). There are no differences between the wild-type controls and the pBri-treated 3xTg mice. There are statistically significant differences between the vehicle control mice and the pBri-treated 3xTg mice and wild-type controls using two-way ANOVA, and the treatment effect had *P* < 0.001. Newman–Keuls *post hoc* analyses indicate that both the wild-type controls and pBri-vaccinated Tg mice were significantly different from the control Tg mice (*P* < 0.01). Tg, transgenic.

Titers for the TgSwDI and 3xTg mice were determined against human purified PHF at Tf for both IgG and IgM and were compared to titers for vehicle-inoculated Tg controls at Tf: TgSwDI IgM (Figure 2A), TgSwDI IgG (Figure 2B), 3xTg IgM (Figure 2C) and 3xTg IgG (Figure 2D). These differences in titer are statistically significant by two-way unpaired *t*-tests (*P* = 0.0038, *P* = 0.0037, *P* = 0.0057 and *P* = 0.04, respectively). Characterization of the PHF preparation used for the Western blot and electron microscopy is shown in Figure 2E and F, respectively.

Figure 2E shows a Western blot of the human PHF sample that was used to seed the ELISA plates shown in Figure 2A to D. For the Western blot, Aβ1-42 (2 μg) was loaded into lane 1 and sonicated human PHF (5 μg) was loaded into lane 2. The membrane of lanes 1 and 2 was developed with PHF1 monoclonal antibodies at a dilution of 1:2000. The film was exposed for 60 sec. No cross-reactivity of the PHF1 antibody with Aβ species can be seen in lane 1. The human PHF preparation consisted of species varying from 50 kDa to more than 200 kDa, plus aggregates of higher molecular weight seen at the top of the gel, as identified by PHF1 in lane 2. Then 5 μg of the same PHF sample were loaded into lanes 3 and 4 and detected by anti-Aβ 4G8 monoclonal antibodies at 1:8000. The membrane shown in lane 3 was exposed for 60 sec, the same duration as the membrane shown in lanes 1 and 2. A very faint band can be seen corresponding to dimeric Aβ, as a minor (<1%) contaminant of the PHF preparation. The membrane shown in lane 4 was loaded with the same PHF as in lane 3 but was exposed for 300 sec to reveal the Aβ species present better. In lane 5, aggregated Aβ1-42 (2 μg) was run and the membrane was detected by 4G8; the film was exposed for 10 sec and shows a monomer band as well as higher molecular weight oligomers. The same PHF sample as run in lanes 2 to 4 was used to coat the ELISA plates that were used in Figures 2A to D at 50 ng/well. The amount of Aβ in the PHF samples run in lanes 2 to 4 was estimated to be approximately 100 pg. This could not be a factor in the ELISA plate reactions shown in Figures 2A to D, since in the ELISA reactions against Aβ peptides shown in Figure 1, no coating below 10 ng/well of Aβ gave results above background. An electron micrograph of the human PHF sample used revealed the expected paired helical filaments (Figure 2F).

### Sensorimotor and cognitive testing

To verify that cognitive testing was not confounded by differences in sensorimotor abilities in the pBri-vaccinated versus control mice, sensorimotor testing was conducted first in both transgenic lines. There were no significant differences between the groups in locomotor activity in terms of distance traveled, maximum speed, mean velocity and rest time for TgSwDI mice (data not shown) or for 3xTg mice (data not shown). Traverse beam testing and rotarod testing also did not reveal any differences between transgenic controls versus treated transgenic mice for both the TgSwDI and 3xTg lines (data not shown).

There were statistically significant differences in the results of the radial arm maze cognitive testing between the untreated control Tg mice versus the treated Tg mice and wild-type controls for TgSwDI mice (Figure 3A) and 3xTg mice (Figure 3B). For TgSwDI mice (Figure 3A) using two-way ANOVA, the treatment effect had *P* < 0.001 and the day effect was not significant. *Post hoc* Newman–Keuls testing indicated that both the wild-type controls and treated Tg mice were significantly different from the control Tg mice (*P* < 0.001). There was no difference between the wild-type controls and the treated Tg mice.

For 3xTg mice (Figure 3B) using two-way ANOVA the treatment effect had *P* < 0.001 and the day effect had *P* < 0.001. *Post hoc* Newman–Keuls testing indicated that both the wild-type controls and treated Tg mice were significantly different from the control Tg mice (*P* < 0.01). There was no difference between the wild-type controls and the treated Tg mice.

### Amyloid quantitation by stereology and biochemical analysis

**Figure 4:**
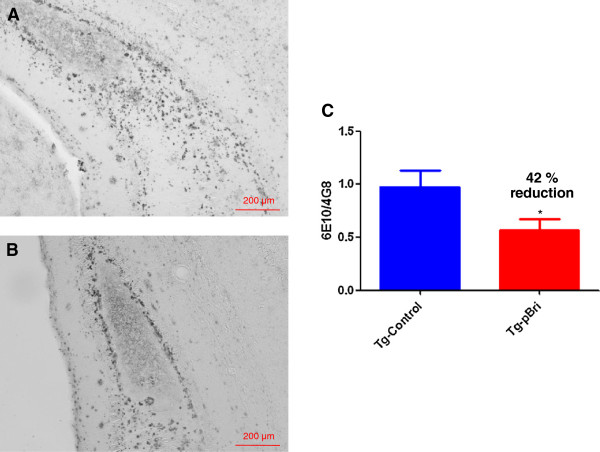
**Quantitation of Aβ burden in the hippocampus of TgSwDI mice.** Representative brain sections of both **(A)** vehicle control and **(B)** pBri-treated Tg mice immunostained with anti-Aβ antibodies 4G8 and 6E10 from the hippocampus. **(C)** Bar graph of amyloid burden calculated using stereology for the hippocampus of vehicle control and pBri-vaccinated TgSwDI mice. On average there was a 42% reduction in the areas occupied by 4G8/6E10 immunoreactivity (*P = 0.039 by an unpaired two-tailed *t*-test). Tg, transgenic.

**Figure 5:**
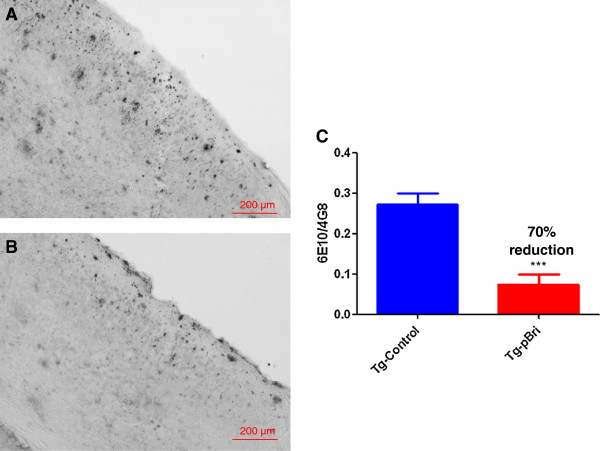
**Quantitation of Aβ burden in the cortex of TgSwDI mice.** Representative brain sections of both **(A)** vehicle control and **(B)** pBri-treated Tg mice immunostained with anti-Aβ antibodies 4G8 and 6E10 from the cortex. **(C)** Bar graph of amyloid burden calculated using stereology for the cortex of vehicle control and pBri-vaccinated TgSwDI mice. On average there was a 70% reduction in the areas occupied by 4G8/6E10 immunoreactivity accounting for precipitated amyloid (***P = 0.0004 by an unpaired two-tailed *t*-test). Tg, transgenic.

**Figure 6:**
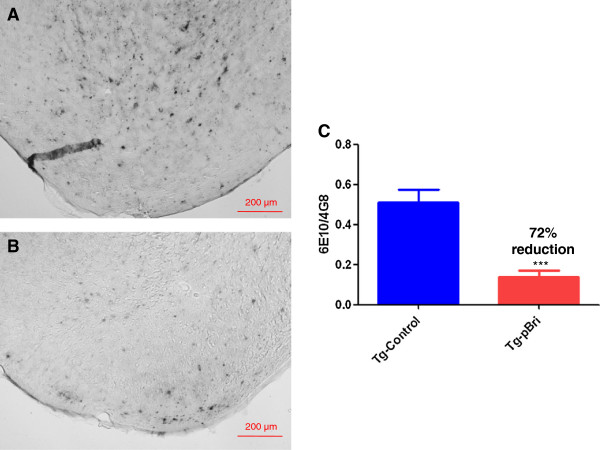
**Quantitation of amyloid burden in the amygdala of TgSwDI mice.** Representative brain sections of both **(A)** vehicle control and **(B)** pBri-treated Tg mice immunostained with anti-Aβ antibodies 4G8 and 6E10 from the amygdala. **(C)** Bar graph of amyloid burden calculated using stereology for the amygdala of vehicle control and pBri-vaccinated TgSwDI mice. On average there was a 72% reduction in the areas occupied by 4G8/6E10 immunoreactivity accounting for precipitated amyloid (*** *P* < 0.0004 by an unpaired two-tailed *t*-test). Tg, transgenic.

There were significant reductions in the amyloid burden (percentage area occupied by 4G8/6E10 immunoreactivity) for the treated TgSwDI mice in the hippocampus (42% reduction, Figure 4), the cortex (70% reduction, Figure 5) and the amygdala (72% reduction, Figure 6) with *P* = 0.039, *P* = 0.0004 and *P* < 0.0004, respectively. Each of the three figures shows a representative section from a control transgenic mouse, a representative section from a pBri-treated transgenic mouse and a histogram of the amyloid burden (scale bar is 200 μm). The amyloid burdens we calculated for these Tg mice are similar to what we and others have previously reported [22,23,29-32]; however, the burdens are less than in some other reports [33]. This variability is likely related to differing experimental procedures between laboratories.

**Figure 7:**
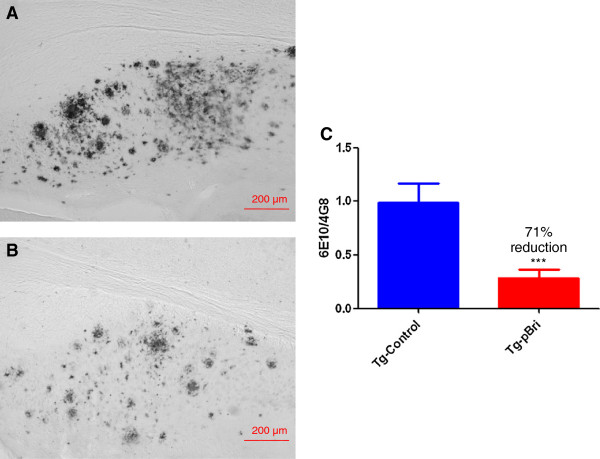
**Quantitation of amyloid burden in the hippocampus of 3xTg mice.** Representative brain sections of both **(A)** vehicle control and **(B)** pBri-treated Tg mice immunostained with anti-Aβ antibodies 4G8 and 6E10 from the hippocampus. **(C)** Bar graph of hippocampus burden calculated using stereology for the hippocampus of vehicle control and pBri-vaccinated 3xTg mice. On average there was a 71% reduction in the areas occupied by 4G8/6E10 immunoreactivity accounting for precipitated amyloid (***P = 0.0006 by an unpaired two-tailed *t*-test). Tg, transgenic.

There were significant reductions in the amyloid burden in the hippocampus of treated 3xTg mice (71% reduction, *P* = 0.0006, Figure 7). Figure 7A is a representation section from a control transgenic mouse. Figure 7B is a representative section from a pBri-treated transgenic mouse and Figure 7C is a histogram of the amyloid burden (scale bar is 200 μm). The amyloid burden in the cortex of these 3xTg mice was too low for meaningful quantitative studies.

**Figure 8:**
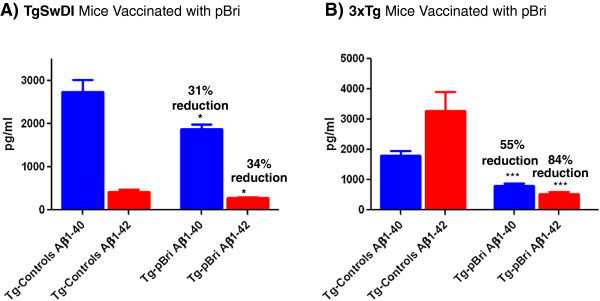
**Levels of soluble Aβ40 and Aβ42 in 20% brain homogenates. (A)** TgSwDI treated and Tg control groups show a 31% and 34% reduction in the levels of Aβ40 (*P = 0.02) and Aβ42 (*P = 0.01). **(B)** There was a 55% and 84% reduction in the levels of Aβ40 (***P < 0.0001) and Aβ42 (***P = 0.0004) between 3xTg treated and vehicle control groups, respectively. *P* values are by unpaired two-tailed *t*-tests. Tg, transgenic.

Significant reductions in the biochemically extracted Aβ40 and Aβ42 levels were also noted for both the TgSwDI and 3xTg mice in the soluble fraction of 20% brain homogenates (Figure 8). For the TgSwDI mice, Aβ40 and Aβ42 levels were reduced by 31% and 34%, respectively (*P* = 0.02 and *P* = 0.01, respectively, by unpaired two-tailed *t*-tests) (Figure 8A). For the 3xTg mice Aβ40 and Aβ42 levels were reduced by 55% and 84%, respectively (*P* < 0.0001 and *P* = 0.0004, respectively) (Figure 8B).

### Quantitation of Aβ oligomers

**Figure 9:**
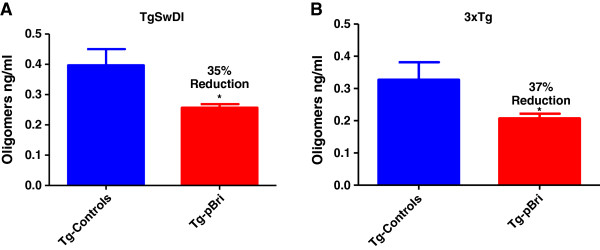
**Quantitation of Aβ oligomers.** Oligomer levels in the soluble brain fractions were determined using an ELISA specific to aggregated/oligomeric Aβ. Two-tail unpaired *t*-tests of this ELISA data show a significant reduction of levels of Aβ aggregates/oligomers for the pBri-treated group vs. the vehicle group for both **(A)** TgSwDI mice (35% reduction) and **(B)** 3xTg mice (37% reduction) (*P = 0.02 and *P = 0.04, respectively, by unpaired two-tailed *t*-tests). Tg, transgenic.

Soluble oligomeric/aggregated Aβ ligands (also known as ADDLs) may account for memory loss and AD neuropathology, thus they are a significant therapeutic target [34]. We measured oligomers in the soluble brain fractions using an ELISA specific to aggregated/oligomeric Aβ, similar to prior studies by us and others [20,35,36]. Two-tail unpaired *t*-tests of this ELISA showed a significant reduction of levels of Aβ aggregates/oligomers for the pBri-treated group vs. the vehicle group for both TgSwDI mice (Figure 9A, 35% reduction) and 3xTg mice (Figure 9B, 37% reduction) (*P = 0.02 and *P = 0.04, respectively, by unpaired two-tailed *t*-tests).

### Quantification of tau burden in 3xTg mice

**Figure 10:**
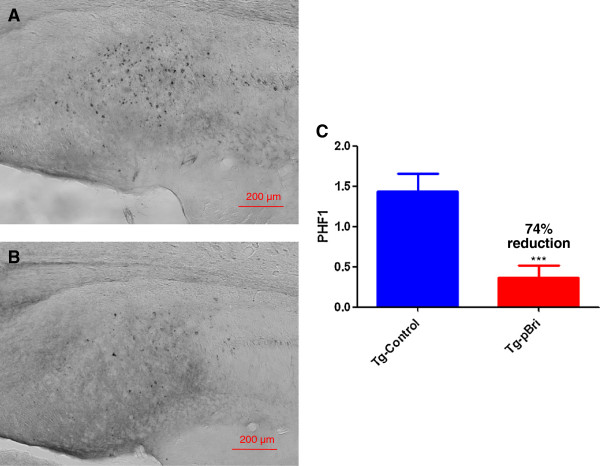
**Quantitation of tau burden using PHF1 for the hippocampus of 3xTg mice.** The tau burden was analyzed semiquantitatively on a scale of 0 to 4. Representative brain sections of both **(A)** vehicle control and **(B)** pBri-treated Tg mice immunostained with PHF1 from the hippocampus. **(C)** Bar graph of tau semiquantitative analysis for the hippocampus of Tg control and pBri-vaccinated mice. On average there was a 74% reduction in the areas occupied by PHF1 immunoreactivity accounting for phosphorylated tau (***P = 0.0005 by an unpaired two-tailed *t*-test). Tg, transgenic.

**Figure 11:**
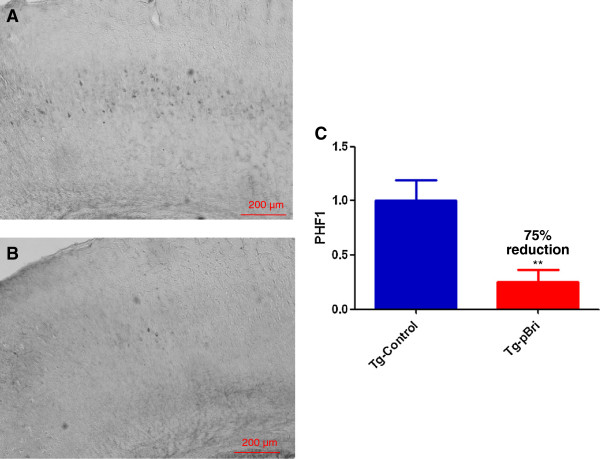
**Quantitation of tau burden using PHF1 for the cortex of 3xTg mice.** The tau burden was analyzed semiquantitatively on a scale of 0 to 4. Representative brain sections of both **(A)** vehicle control and **(B)** pBri-treated Tg mice immunostained with PHF1 from the cortex. **(C)** Bar graph of tau semiquantitative analysis for the cortex of Tg control and pBri-vaccinated mice. On average there was a 75% reduction in the areas occupied by PHF1 immunoreactivity accounting for phosphorylated tau (***P = 0.0017 by an unpaired two-tailed *t*-test). Tg, transgenic.

**Figure 12:**
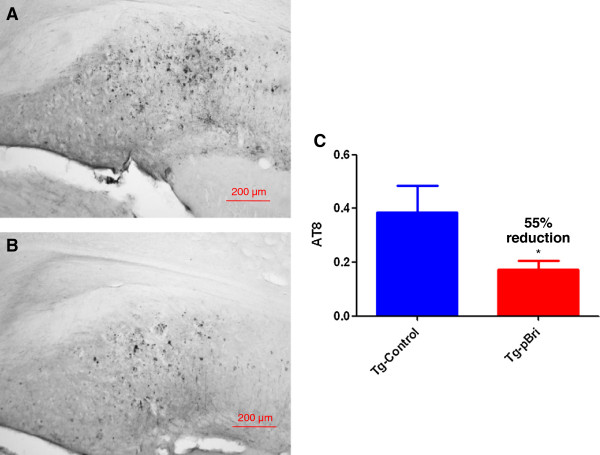
**Quantitation of tau burden using AT8 for the hippocampus of 3xTg mice.** The tau burden was analyzed semiquantitatively on a scale of 0 to 4. Representative brain sections of both **(A)** vehicle control and **(B)** pBri-treated Tg mice immunostained with AT8 from the hippocampus. **(C)** Bar graph of tau semiquantitative analysis for the hippocampus of Tg control and pBri-vaccinated mice. On average there was a 55% reduction in the areas occupied by AT8 immunoreactivity accounting for phosphorylated tau (*P = 0.03 by an unpaired two-tailed *t*-test). Tg, transgenic.

To determine the treatment effect of pBri immunomodulation on tau pathology for 3xTg mice, we used a semiquantitative analysis to quantify the tau burden in the region of the hippocampus and cortex for the serial sections as we have previously published [20]. Two anti-tau antibodies, AT8 (which recognizes tau phosphorylated at both serine 202 and threonine 205) and PHF1 (which recognizes phospho-tau epitopes serine 396 and 404), were used. AT8 immunoreactivity was observed mainly in the hippocampus of our 3xTg mice, while PHF1 immunoreactivity was observed in both the hippocampus and cortex. PHF1 immunoreactivity was reduced by 74% and 75% in the hippocampus (Figure 10) and cortex (Figure 11) of pBri-vaccinated mice, respectively (*P* = 0.0005 and *P* = 0.0017 by two-tailed *t*-tests, respectively). AT8 immunoreactivity was reduced by 55% in the hippocampus of pBri-treated mice (*P* = 0.03) (Figure 12).

### Quantitation of phosphorylated tau in the soluble brain homogenate by Western blot using PHF1 and Meso Scale Discovery analysis of total tau and phosphorylated tau (Thr231)

**Figure 13:**
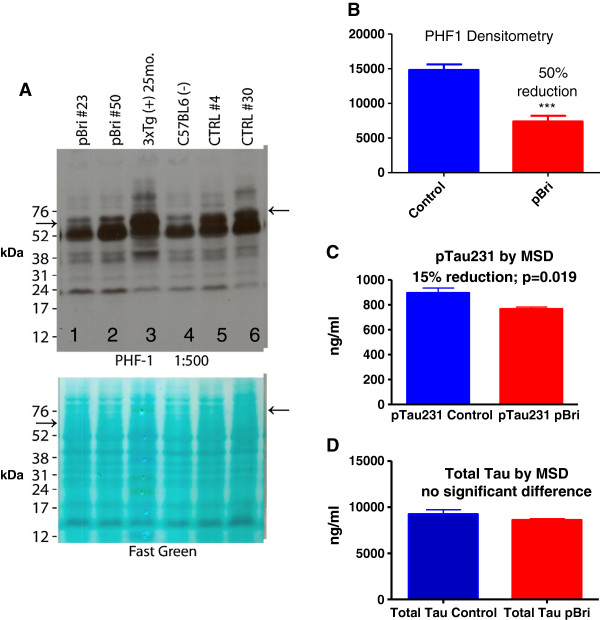
**Quantitation of phosphorylated tau in the soluble brain homogenate by Western blot using PHF1 for 3xTg mice. (A)** Upper: Western blot using PHF1. Lower: Membrane stained with Fast Green before being probed with PHF1, shown to illustrate that there was an equal protein load in each of the lanes. **(B)** Densitometric quantitation of the specific, approximately 60 kDa, PHF1 immunoreactive band, with a 50% reduction for the pBri-treated 3xTg mice (*P* < 0.0001 by an unpaired two-tailed *t*-test). **(C)** Quantitation of phosphorylated tau (Thr231) and **(D)** quantitation of total tau using a Meso Scale Discovery (MSD) system that utilizes electrochemiluminescence analysis. There is a significant reduction of phosphorylated tau (Thr231; *P* = 0.019 by two-tailed *t*-test) of 15%. There is no significant difference in total tau when comparing pBri-immunized and control 3xTg mice. MSD, Meso Scale Discovery.

A PHF1-specific band was noted at approximately 60 kDa by Western blotting (see arrows in Figure 13A). This band has been previously reported in 3xTg mice using PHF1; other bands were also detected but are not specific for phosphorylated tau [37]. Densitometric quantitation of this band showed there was a 50% reduction for the pBri-treated 3xTg mice (see Figure 13B, *P* < 0.0001 by an unpaired two-tailed *t*-test). Figure 13A shows representative samples from two pBri-treated mice in lanes 1 and 2. A sample from a 25-month-old 3xTg mouse (with more extensive tau pathology) was run as a positive control in lane 3. In lane 4, a sample from an age-matched wild-type mouse was run as a negative control, highlighting that the other bands seen with PHF1 are not specific for phosphorylated tau. Representative samples from two vehicle-treated 3xTg control mice were run in lanes 5 and 6. A strong band can be seen at approximately 60 kDa in lanes 5 and 6 (see arrow). This band has about the same intensity as the equivalent band for the positive control, the 25-month-old 3xTg mouse sample in lane 3, and is much stronger than the equivalent bands for the pBri-treated mice in lanes 1 and 2. The membrane stained with Fast Green shows there was an equal protein load in each of the lanes.

MSD analysis of phosphorylated tau (Thr231) showed a significant reduction (*P* = 0.019) of 15% (Figure 13C), while the analysis of total tau showed there was no significant difference between control and pBri-treated 3xTg mice (Figure 13D).

### Assessment of astrocytosis and microglial activation

**Figure 14:**
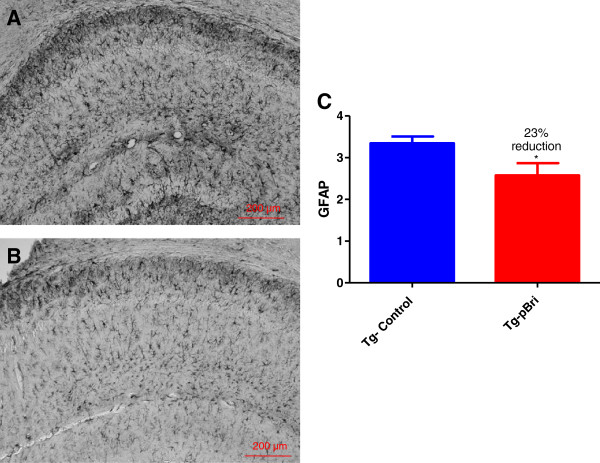
**Quantitation of astrocytosis in the hippocampus of TgSwDI mice.** Reactive astrocytosis was analyzed semiquantitatively on a scale of 0 to 4. Representative brain sections of both **(A)** vehicle control and **(B)** pBri-treated Tg mice immunostained with GFAP from the hippocampus. **(C)** Bar graph of astrocyte semiquantitative analysis in the hippocampus for vehicle control and pBri-vaccinated mice. On average there was a 23% reduction in the areas occupied by GFAP immunoreactivity accounting for astrocytosis (*P = 0.03 by an unpaired two-tailed *t*-test). GFAP, glial fibrillary acidic protein; Tg, transgenic.

**Figure 15:**
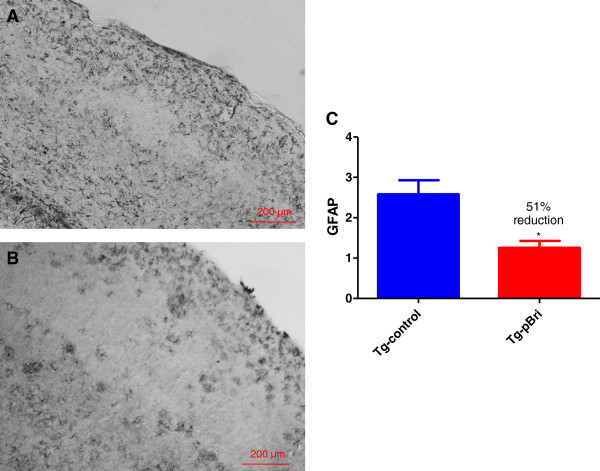
**Quantitation of astrocytosis in the cortex of TgSwDI mice.** Reactive astrocytosis was analyzed semiquantitatively on a scale of 0 to 4. Representative brain sections of both **(A)** vehicle control and **(B)** pBri-treated Tg mice immunostained with GFAP from the cortex. **(C)** Bar graph of astrocyte semiquantitative analysis for the cortex of vehicle control and pBri-vaccinated mice. On average there was a 51% reduction in the areas occupied by GFAP immunoreactivity accounting for astrocytosis (*P = 0.015 by an unpaired two-tailed *t*-test). GFAP, glial fibrillary acidic protein; Tg, transgenic.

**Figure 16:**
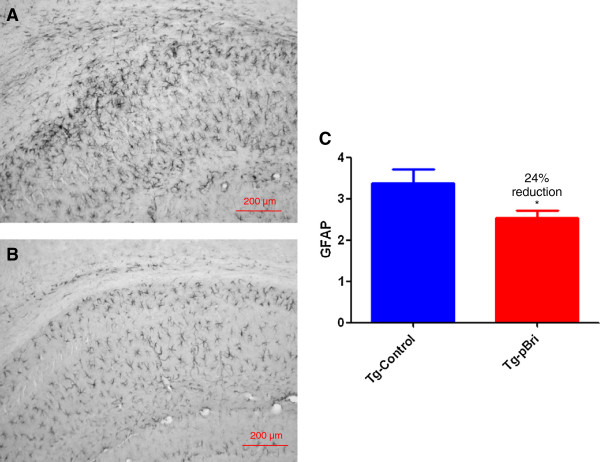
**Quantitation of astrocytosis in the hippocampus of 3xTg mice.** Reactive astrocytosis was analyzed semiquantitatively on a scale of 0 to 4. Representative brain sections of both **(A)** vehicle control and **(B)** pBri-treated Tg mice immunostained with GFAP from the hippocampus. **(C)** Bar graph of astrocyte semiquantitative analysis for the hippocampus of vehicle control and pBri-vaccinated mice. On average there was a 24% reduction in the areas occupied by GFAP immunoreactivity accounting for astrocytosis (*P = 0.03 by an unpaired two-tailed *t*-test). GFAP, glial fibrillary acidic protein; Tg, transgenic.

**Figure 17:**
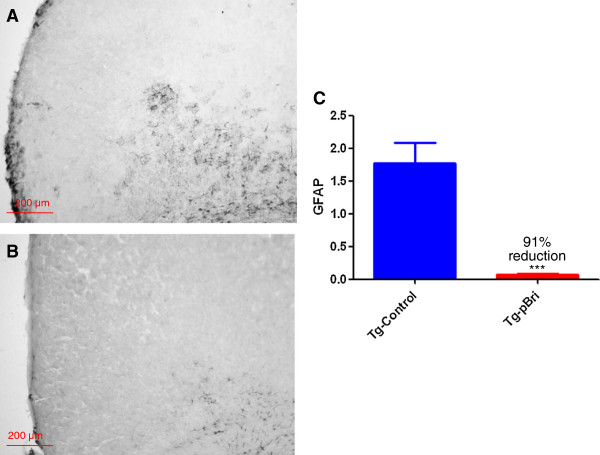
**Quantitation of microgliosis in the cortex of 3xTg mice.** Microglial burden was analyzed semiquantitatively on a scale of 0 to 4. Representative brain sections of both **(A)** vehicle control and **(B)** pBri-treated Tg mice immunostained with CD45 from the cortex. **(C)** Bar graph of microglia semiquantitative analysis for the cortex of vehicle control and pBri-vaccinated mice. On average there was a 91% reduction in the areas occupied by CD45 immunoreactivity accounting for microgliosis (***P = 0.0001 by an unpaired two-tailed *t*-test). GFAP, glial fibrillary acidic protein; Tg, transgenic.

GFAP immunoreactivity in the hippocampus (Figure 14) and cortex (Figure 15) for pBri-treated TgSwDI mice versus control Tg mice was reduced by 23% and 51%, respectively (*P* = 0.03 and *P* = 0.015 by two-tailed unpaired *t*-tests). GFAP immunoreactivity in the hippocampus (Figure 16) and cortex (Figure 17) for pBri-treated 3xTg mice versus control Tg mice was reduced significantly by 24% and 91%, respectively (*P* = 0.03 and *P* = 0.0001 by two-tailed unpaired *t*-tests).

**Figure 18:**
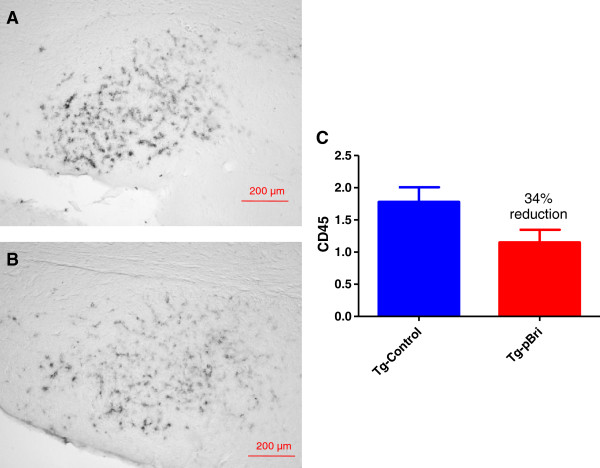
**Quantitation of microglial burden in the hippocampus of TgSwDI mice.** Microglial burden was analyzed semiquantitatively on a scale of 0 to 4. Representative brain sections of both **(A)** vehicle control and **(B)** pBri-treated Tg mice immunostained with CD45 from the hippocampus. **(C)** Bar graph of microglia semiquantitative analysis for the hippocampus of vehicle control and pBri-vaccinated mice. On average there was a 34% reduction in the areas occupied by CD45 immunoreactivity accounting for microgliosis (*P* < 0.05 by an unpaired one-tailed *t*-test). Tg, transgenic.

**Figure 19:**
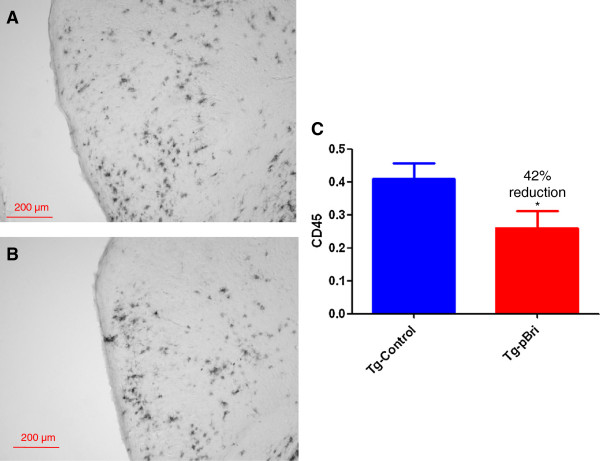
**Quantitation of microglial burden in the cortex of TgSwDI mice.** Microglial burden was analyzed semiquantitatively on a scale of 0 to 4. Representative brain sections of both **(A)** vehicle control and **(B)** pBri-treated Tg mice immunostained with CD45 from the cortex. **(C)** Bar graph of microglia semiquantitative analysis for the cortex of vehicle control and pBri-vaccinated mice. On average there was a 42% reduction in the areas occupied by CD45 immunoreactivity accounting for microgliosis (*P = 0.027 by an unpaired one-tailed *t*-test). Tg, transgenic.

**Figure 20:**
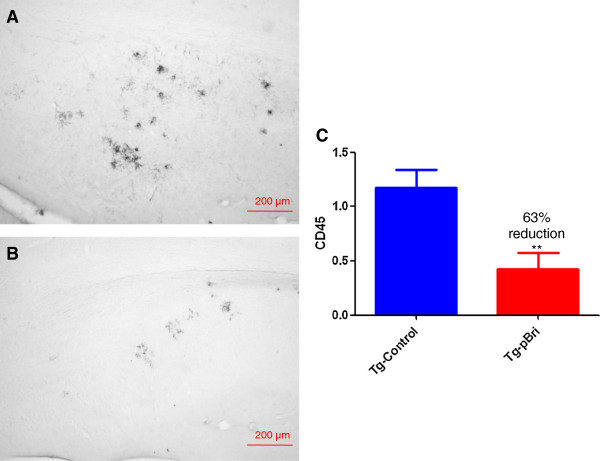
**Quantitation of microglial burden in the hippocampus of 3xTg mice.** Microglial burden was analyzed semiquantitatively on a scale of 0 to 4. Representative brain sections of both **(A)** vehicle control and **(B)** pBri-treated Tg mice immunostained with CD45 from the hippocampus. **(C)** Bar graph of microglia semiquantitative analysis for the hippocampus of vehicle control and pBri-vaccinated mice. On average there was a 63% reduction in the areas occupied by CD45 immunoreactivity accounting for microgliosis (**P = 0.0035 by an unpaired two-tailed *t*-test). Tg, transgenic.

The assessment of the microglial marker CD45 was based on semiquantitative analyses of the extent of microgliosis, as we have previously reported [20,22]. pBri treatment resulted in an overall hippocampal (Figure 18) and cortical (Figure 19) reduction in CD45 immunoreactivity for TgSwDI mice of 34% and 42%, respectively (*P* < 0.05 and *P* = 0.027 by unpaired one-tailed *t*-tests). For 3xTg mice, pBri treatment resulted in a 63% reduction of CD45 immunoreactivity in the hippocampus (Figure 20, *P* = 0.0035 by an unpaired two-tailed *t*-test). CD45 immunoreactivity in the cortex of 3xTg mice was too low for quantitation.

### Quantitation of microhemorrhages by Perl stain in TgSwDI mice

**Figure 21:**
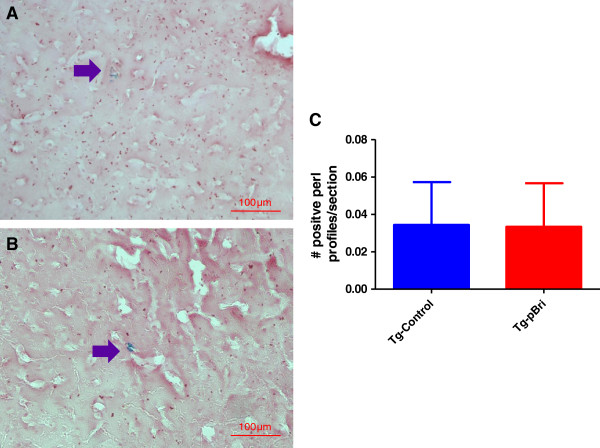
**Quantitation of microhemorrhaging in the cortex and hippocampus of TgSwDI mice.** The number of positive profiles were quantified per section. Representative Perl-stained brain sections of both **(A)** vehicle control and **(B)** pBri-treated Tg mice are shown. Arrows point to Perl positive profiles. **(C)** Bar graph of Perl stain semiquantitative analysis depicts no significant difference between Tg controls and pBri-treated 3xTg mice. Tg, transgenic.

The number of microhemorrhages in TgSwDI mice was evaluated by Perl staining as we have previously published [22]. No significant differences in microhemorrhages were noted in the pBri-treated TgSwDI mice versus control vehicle-treated Tg mice (Figure 21).

## Discussion

We demonstrated that immunization with a non-self-peptide in a polymerized, β-sheet-rich form in two transgenic mice lines, which model either vascular amyloid or tau-related pathology, is able through conformational mimicry to induce an immune response to both pathological Aβ42 and PHF. This extends our previous results that our novel immunomodulatory approach is able to reduce amyloid plaque pathology in APP/PS1 Tg mice [16]. Hence, we now show that active immunization with a non-self-antigen in a conformation that is oligomeric can be effective at ameliorating all the cardinal lesions of AD, namely amyloid plaques, congophilic amyloid angiopathy and neurofibrillary tangles, without apparent toxicity. The immunogen we used corresponds to the 13 amino acids of the carboxyl end of the amyloid that is deposited in British amyloidosis, where a missense mutation in a stop codon results in the transcription of a novel intronic sequence [14,15,38]. This peptide has no homology to known mammalian proteins, but is highly amyloidogenic [15,39]. This immune response resulted in a clear behavioral rescue in treated TgSwDI and 3xTg mice as they performed similarly to wild-type mice in the radial arm maze. The testing of this cognitive task was not confounded by any differences in the sensorimotor activity between the control and vaccinated mice as shown by the locomotor activity, rotarod and traverse beam testing, where there were no significant differences between treated and control Tg mice for both mouse lines. This behavioral rescue was associated with a marked reduction in Aβ-related pathology as determined histologically and biochemically. The anti-Aβ42 and anti-PHF titers our vaccinated mice developed were relatively modest; however, as we and others have suggested, behavioral rescue is most closely linked to oligomer reduction and not with either the degree of amyloid plaque deposition reduction or overall anti-Aβ titer [40-42]. Hence, it is not the absolute degree of the humoral response generated to Aβ or tau epitopes but its quality in terms of effective targeting of toxic species that is likely the most important factor.

The relatively modest titers also reflect two important facts about our approach. First, we made a deliberate choice of aluminum hydroxide (alum) as an adjuvant. Alum is a relatively mild adjuvant but it is approved for human use and is widely used [43]. The majority of immunization studies in AD mouse models have used much stronger adjuvants that are not approved for use in humans [32] and also produce a cytotoxic-prone immune response; hence, the results are more difficult to translate to future human trials. Second, the most toxic forms of both Aβ and tau are thought to be oligomeric [9,34]. Importantly we showed that our approach (despite the relatively low titers) was associated with a reduction of these toxic oligomer species, as documented by different assays, one specific to aggregated Aβ species (Figure 9) and others against phosphorylated tau species (Figure 13). This is also consistent with our finding using ELISA for the brain soluble fraction that levels of total Aβ42 species were more significantly reduced compared to total Aβ40 species. It is well known that Aβ42 species are more prone to form oligomers and fibrils. This more specific targeting of Aβ42 species was most notable for the 3xTg mice where Aβ42 levels were reduced by 84%, compared to a 55% reduction of Aβ40 species (see Figure 8). In addition we demonstrated biochemically that abnormally phosphorylated tau species identified by PHF1 (serine 396 and 202) and by MSD (Thr 231) were reduced by approximately 50% and 15%, respectively, in the brain soluble fraction, the fraction which contains tau oligomers (Figure 13). Therefore we document the reduction of both Aβ and tau oligomeric species by our immunomodulatory approach. The reduction of tau pathology is likely to be the result of a combination of the direct effects of the sustained conformational anti-PHF immune response to oligomeric forms of tau that we found (see Figures 2 and 13), as well as an indirect effect by reducing Aβ oligomers in the pBri 3xTg immunized mice.

The majority of previous active and passive immunization studies in mouse models and all the previous trials in humans have targeted both the normal conformer and the pathological conformer of either Aβ or tau [44]. This can be problematic as targeting normal sAβ may inhibit its known physiological functions such as neuroprotection, modulation of long-term potentiation and innate immunity, and there is the risk of inducing autoimmune complications [45-47]. The targeting of tau by active or passive immunization also carries the risk of neurotoxicity and has an encephalogenic potential [44,48]. Our immunomodulation approach greatly reduces the chance of such toxicity as the immunogen is a non-self-peptide in an oligomeric conformation. In an initial human trial of active vaccination, the use of a self-antigen as an immunogen (Aβ42) was associated with encephalitis in some 6% of patients [49], which was linked to excess cell-mediated inflammation [44,50].

An additional autoimmune complication in ongoing human trials of passive immunization using anti-Aβ antibodies has been vasogenic edema or amyloid-related imaging abnormalities (ARIA), a complication that is particularly common in apoE4 carriers [51]. The etiology of ARIA is thought to be directly linked to the removal of amyloid from CAA lesions in blood vessel walls, rendering them more likely to undergo leakage or hemorrhage [52]. CAA occurs in about 98% of AD patients with approximately 75% of these cases rated as severe CAA [53-55]. In addition, CAA is present in about 30% of non-demented elderly individuals [53]. Clinical studies have shown a strong correlation between cognitive impairment and the presence of CAA [56-58]. Furthermore, CAA is associated with focal ischemia and cerebral hemorrhage due to weakening of vascular walls by amyloid deposits and focal inflammation. Hence, it is important that we found a marked reduction of CAA pathology in TgSwDI mice from 42% (in the hippocampus) to 72% (in the amygdala), without an increase in microhemorrhages or inflammatory toxicity. A caveat is that CAA in TgSwDI mice is primarily microvascular, whereas in AD patients amyloid is also found in larger vessels; however, these microvascular amyloid deposits are also associated with hemorrhages and correlate more closely with the presence of dementia [59]. In both TgSwDI and 3xTg mice there were significant reductions of astrocytosis and microgliosis as seen by GFAP and CD45 immunoreactivity. Hence our active immunization approach, which specifically targets pathological conformers only, is effective at lesion reduction without apparent autoimmune complications. It is also notable that our immunization approach uses alum as an adjuvant. Alum has been the most widely used human adjuvant for over 80 years and is regarded as very safe [43]. It predominantly stimulates humoral immunity, and is much less likely to be associated with cytotoxic T-cell responses, which have been linked to toxicity in human AD vaccination trials [3,43]. Our use of alum as an adjuvant facilitates the ease with which our approach could be translated into human trials.

Another significant drawback of the current immunization approaches being tested in humans is that they target only Aβ-related pathology. So far none of these trials has seen a robust clinical improvement, leading some to question the amyloid cascade hypothesis [13,60,61]. The limited autopsy data from the initial human active vaccination trial targeting Aβ42 showed that patients had partial or near complete plaque removal and a reduction of Aβ load compared to age-matched non-immunized controls. However, there were no differences between placebo and active immunization groups in the long-term survival outcome, time to severe dementia or in cognitive outcome measurements [60]. Among living patients taking part in a passive immunization trial targeting Aβ, a 25% amyloid reduction versus controls was documented using PET imaging studies, in the absence of measurable cognitive benefits [61]. It has been suggested that the failure to produce clear clinical benefits is related to therapy being started too late in the very long process of AD pathology accumulation. Aβ and tau-related pathology begins to develop up to approximately 25 years before the onset of obvious clinical signs of even mild cognitive impairment [62]. Hence, Aβ-directed immunotherapy would have to start many years before the patient is symptomatic to have a greater likelihood of being highly efficacious. This suggests that for an immunotherapeutic approach to be effective in symptomatic AD, the intervention should concurrently target both Aβ and tau toxic conformers specifically. There is a growing consensus regarding the importance of specifically targeting both tau and Aβ soluble aggregates therapeutically [8,9]. We reported the first active immunization approach that could achieve this goal [16]. Subsequently, another group has developed an analogous approach using a random sequence amyloid oligomer mimetic peptide, confirming the validity of this approach, at least in AD animal models [63,64].

In summary, we found that a novel active immunization approach, using pBri in an oligomer conformation, is able to ameliorate all the cardinal lesions of AD pathology, amyloid plaques, CAA and NFTs, without apparent toxicity in different animal models that illustrate each of these pathologies. Approaches that target pathological protein conformation have the potential to be effective in a large spectrum of neurodegenerative diseases.

## Abbreviations

Aβ: amyloid β; ABri: British amyloidosis; AD: Alzheimer’s disease; ARIA: amyloid-related imaging abnormalities; BBS: borate buffer saline; Bri: peptide corresponding to the 13 amino acids of the carboxyl end of ABri; BSA: bovine serum albumin; CAA: congophilic amyloid angiopathy; ECL: enhanced chemiluminescence; EDTA: Ethylenediaminotetraacetic acid disodium salt; EGTA: Ethylene glycol tetraacetic acid tetrasodium salt; GFAP: glial fibrillary acidic protein; MSD: Meso Scale Discovery; NFT: Neurofibrillary tangles; OD: optical density; pBri: polymerized British amyloidosis carboxi-terminal 13 amino acid peptide; PHF: paired helical filaments; SDS: sodium-dodecyl sulfate; TBS-T: 50 mM tris-buffer-saline- 0.1% Tween 20; Tg: transgenic; TMB: tetramethylbenzidine.

## Competing interests

The authors declare that they have no competing interests.

## Authors’ contributions

FG and TW designed the experiments, analyzed the results and wrote the paper. FG carried out the antigen preparations, and animal inoculations. KH participated in all the histochemical experiments and helped prepare figures. DP participated in biochemical experiments, ELISAs, blots and helped design some of the figures. KW participated in ELISAs, blots, immunohistochemistry, biochemistry and some behavioral testing. YJ carried out most of the sensorimotor, all locomotor and radial arm maze experiments. YS took care of the experimental animals during the course of the experiments and tested the transgenic status of the animals. PM performed Aβ peptide level analysis. TW supervised all experiments. All authors read and approved the final manuscript.
